# Estradiol-independent restoration of T-cell function in post-reproductive females

**DOI:** 10.3389/fendo.2023.1066356

**Published:** 2023-01-23

**Authors:** Tristin L. King, B. Shaun Bryner, Kaden B. Underwood, McKenna R. Walters, Shawn M. Zimmerman, Nathan K. Johnson, Jeffrey B. Mason

**Affiliations:** ^1^ Department of Animal, Dairy and Veterinary Sciences, Center for Integrated BioSystems, College of Veterinary Medicine, Utah State University, Logan, UT, United States; ^2^ Utah Veterinary Diagnostic Laboratory, College of Veterinary Medicine, Utah State University, Logan, UT, United States

**Keywords:** immune, T-cell, ovarian, hormones, health span

## Abstract

Aging leads to a general decline in protective immunity. The most common age-associated effects are in seen T-cell mediated immune function. Adult mice whose immune systems show only moderate changes in T-cell subsets tend to live longer than age-matched siblings that display extensive T-cell subset aging. Importantly, at the time of reproductive decline, the increase in disease risks in women significantly outpace those of men. In female mice, there is a significant decline in central and peripheral naïve T-cell subsets at the time of reproductive failure. Available evidence indicates that this naïve T-cell decline is sensitive to ovarian function and can be reversed in post-reproductive females by transplantation of young ovaries. The restoration of naïve T-cell subsets due to ovarian transplantation was impressive compared with post-reproductive control mice, but represented only a partial recovery of what was lost from 6 months of age. Apparently, the influence of ovarian function on immune function may be an indirect effect, likely moderated by other physiological functions. Estradiol is significantly reduced in post-reproductive females, but was not increased in post-reproductive females that received new ovaries, suggesting an estradiol-independent, but ovarian-dependent influence on immune function. Further evidence for an estradiol-independent influence includes the restoration of immune function through the transplantation of young ovaries depleted of follicles and through the injection of isolated ovarian somatic cells into the senescent ovaries of old mice. While the restoration of naïve T-cell populations represents only a small part of the immune system, the ability to reverse this important functional parameter independent of estradiol may hold promise for the improvement of post-reproductive female immune health. Further studies of the non-reproductive influence of the ovary will be needed to elucidate the mechanisms of the relationship between the ovary and health.

## Background

In human females, a strong relationship exists between chronological life span, reproductive life span and health. Chronological life span in humans has been expanded dramatically over the last century (1), but over the same period, the timing of menopause or the end of the reproductive life span has remained relatively constant (2). Prior to ovarian failure, females hold a significant health advantage over males of the same age. However, increases in disease risks in women at the time of reproductive decline significantly outpace those of men (3). This connection between reproductive function and the maintenance of health is magnified in women with premature ovarian failure, who suffer from increased rates of disease at a much younger age than in women with traditional menopausal timing (4). Ovarian failure prior to 40 years of age can sharply increase mortality rates (5, 6), compared with women that report natural menopause at ages 50–54 (7–9).

Aging also leads to a general decline in protective immunity. Immunosenescence is a general decline in the overall function of the immune system, which often results in poor responses to vaccines, the emergence of latent diseases and increased duration and/or severity of illness after infection (10). The most common effects are usually in T-cell-mediated immune function, which can be a result of aging-related changes in many cells and tissues, including a decline in thymic function and thymic involution/atrophy. T-cell-mediated immune function contributes to many aspects of defense against viral and microbial infections. There is strong evidence for an age-associated decline in the naïve subset of T-cells that have not been exposed to a stimulatory antigen, and a consequent age-associated increase in memory T-cells with at least one cycle of antigen-stimulated activation and proliferation (11, 12).

Having an adequate number of naïve T-cells is crucial for the immune system to respond to novel pathogens. Changes in T-cell subsets are often thought to be gender-specific and influenced by a potential connection between sex hormones and the immune system. While there are not many studies exploring gender-specific immunosenescence, in the female mice we study, there is a significant decline in CD4+ and CD8+ central and peripheral naïve T-cells at the time of reproductive failure (13, 14).

While the value of ovarian hormones in female health is unquestionable, efforts to replace the hormonal milieu of actively cycling ovaries in peri- and post-menopausal women have struggled to reliably restore the health benefits enjoyed by young, reproductively cycling women with young ovaries. Menopausal ovarian failure is tied to the loss of ovarian oocytes. Hypophysectomy in the mouse significantly retards, but does not prevent, the loss of oocytes from the ovary (15, 16). The aging process, as it affects the ovary of the mouse, is therefore retarded when the pituitary is removed. Immunological function peaks at puberty, declines by 50% during the post-reproductive life span in mice and humans (17, 18). In the adult female rat, 8 weeks after ovariectomy, atrophy of the thyroid was apparent along with hypertrophy of the pituitary (19, 20), supporting the role of the ovary in immune function.

Our studies with normal, non-transgenic aged mice demonstrated that transplanting young ovaries into old mice increases health and lifespan after ovarian transplant. These novel findings indicate that young ovarian tissue can provide factors that slow/reverse the aging process. Prevailing views speculate that the positive effects of transplanting young ovaries into old mice are due to restoration of cyclic hormonal activity. However, we showed that depleting the hormone-regulating follicles prior to transplantation also increased health and produced an even greater lifespan extension in recipient animals. Additionally, our most recent study with transplantation of isolated young ovarian somatic cells to old endogenous ovaries revealed that these cells alone are sufficient for extension of health span. This strongly suggests that additional factors other than ovarian germ cells and/or cyclic ovarian steroids are responsible for the health benefits of ovarian tissue. Here, we specifically examined the influence of young ovarian tissues on immune function in post-reproductive female mice in the presence or absence of ovarian follicular influence.

## Influence of age and ovarian function on T-cell subsets

The evidence supporting an age-associated decline in the naïve subset of T-cells and a consequent decrease in the ratio of naïve-to-memory T-cells is robust (21, 22). Adult mice with only moderate changes in T-cell subsets live longer than age-matched siblings that have more extensive T-cell subset aging (23). We previously reported a significant, age-associated decline in both CD4+ and CD8+ central and peripheral (effector) naïve T-cells from 6 to 16 months of age in CBA/J female mice (CBA/J female mice normally become reproductively senescent at approximately 11 months of age, 13, 24, **Table 1**). The change in the naïve: memory T-cell ratios was predominately due to a change in naïve subsets and not memory subsets, as both CD4+ and CD8+ central and peripheral memory T-cells did not change or tended to slightly increase during the period from 6 to 16 months. In addition, the ratio of CD4+ to CD8+ cells declined in parallel to the decline in naïve cells. More recent T-cell data from older mice (21mo) suggests that the trends established to 16 months continue until very old age (mean age at death is 644d/21mo in CBA/J female mice) and that the effects on CD8+ cells, which appear somewhat delayed, compared to the effects on CD4+ cells to 16 months, are accelerated at advanced ages. The influence on T-cell subsets seen in CBA/J female mice was also seen in C57BL/6 female mice, albeit at slightly older ages. (C57BL/6 female mice in our colony are normally reproductively competent at 11 months of age). The pathology reports for aged mice normally listed the thymus as involuted/consisting of fat/missing (Dr. Stephen Griffey, School of Veterinary Medicine, University of California, Davis and Dr. Yuji Ikeno, Barshop Institute for Longevity and Aging Studies and Department of Pathology at UTHSCSA). Thymic involution, which is directly related to T-cell development is exacerbated at puberty, slowed by castration and is accelerated by exogenous estradiol (25). Our FD and OSC recipients were not cycling and none of our recipient mice demonstrated changes in estradiol from age-matched controls. Our pathology reports for all aged mice normally listed the thymus as involuted/consisting of fat/missing in all old groups regardless of treatment, suggesting an extra-thymic mechanism of T-cell influence in transplant recipients.

**Table 1:** Percent change from 6 month old control mice-aging/treatment effects.

	CD4 C Naive/memory	CD4 P Naive/memory	CD8 C Naive/memory	CD8 P Naive/memory	CD4/CD8
6mo CTL	100%	100%	100%	100%	100%
11mo CTL	51%	29%	103%	65%	72%
19mo CTL	18%	7%	28%	20%	49%
29mo CTL	16%	7%	16%	7%	38%
21mo FC	41%	25%	47%	37%	90%
21mo FD	58%	28%	56%	43%	67%
19moOSC	71%	64%	72%	33%	79%

CTL, control mice; FC, transplanted with follicle-containing young 60d ovaries at 13mo of age; FD, transplanted with follicle-depleted young 60d ovaries at 13mo of age; OSC, transplanted with FC, transplanted with follicle-containing young 60d ovaries at 13mo of age; FD, transplanted with follicle-depleted young 60d ovaries at 13mo of age; OSC, transplanted with isolated young isolated young 60d ovarian somatic cells at 13mo of age.

What is often viewed as an inevitable increase in disease associated with the menopausal transition may be more malleable than formerly thought. The replacement of young ovarian tissues in old, reproductively-senescent female mice has demonstrated significant restoration of many health span parameters (13, 14, 26–35). The naïve T-cell decline seen in control mice (**Table 1**) was strongly reversed in 16-month-old females by ovarian transplantation with young, 60d transplanted ovaries at 11 months of age (13, **Table 2**). In this scenario, both 7-month-old control females and 16-month-old transplant recipients possessed 7-month-old ovaries. Interestingly, the pathology reports for the thymus of aged, transplant recipient mice were similar to untreated controls and were most often listed as consisting of fat/missing. The restoration of naïve T-cell subsets by ovarian transplantation was dramatic, but represented only a limited restoration of T-cell function of that seen at 6 months of age. Therefore, the ovarian-dependent change in immune function was likely an indirect effect moderated by other physiological functions. Based on current dogma, a likely ‘other physiological function’ would be the hormone estradiol.

**Table 2:** Percent decrease of T cell ratios from 6 month mice-treatment effect.

	CD4 C Naïve/memory	CD4 P Naïve/memory	CD8 C Naïve/memory	CD8 P Naïve/memory	CD4/CD8
19mo CTL	>75%	>75%	>50%	>75%	>50%
21mo FC	>25%	>50%	>50%	>25%	<25%
21mo FD	>25%	>50%	>25%	>25%	>25%
19moOSC	>25%	>25%	>25%	>25%	<25%

FC, transplanted with follicle-containing young 60d ovaries at 13mo of age; FD, transplanted with follicle-depleted young 60d ovaries at 13mo of age; OSC, transplanted with FC, transplanted with follicle-containing young 60d ovaries at 13mo of age; FD, transplanted with follicle-depleted young 60d ovaries at 13mo of age; OSC, transplanted with isolated young isolated young 60d ovarian somatic cells at 13mo of age.

Following this dogma, we originally hypothesized that this restoration of naïve T-cells was driven by germ cell-stimulated ovarian hormone production from the young, transplanted ovaries.

## Ovarian function and estradiol

**Figure 1:**
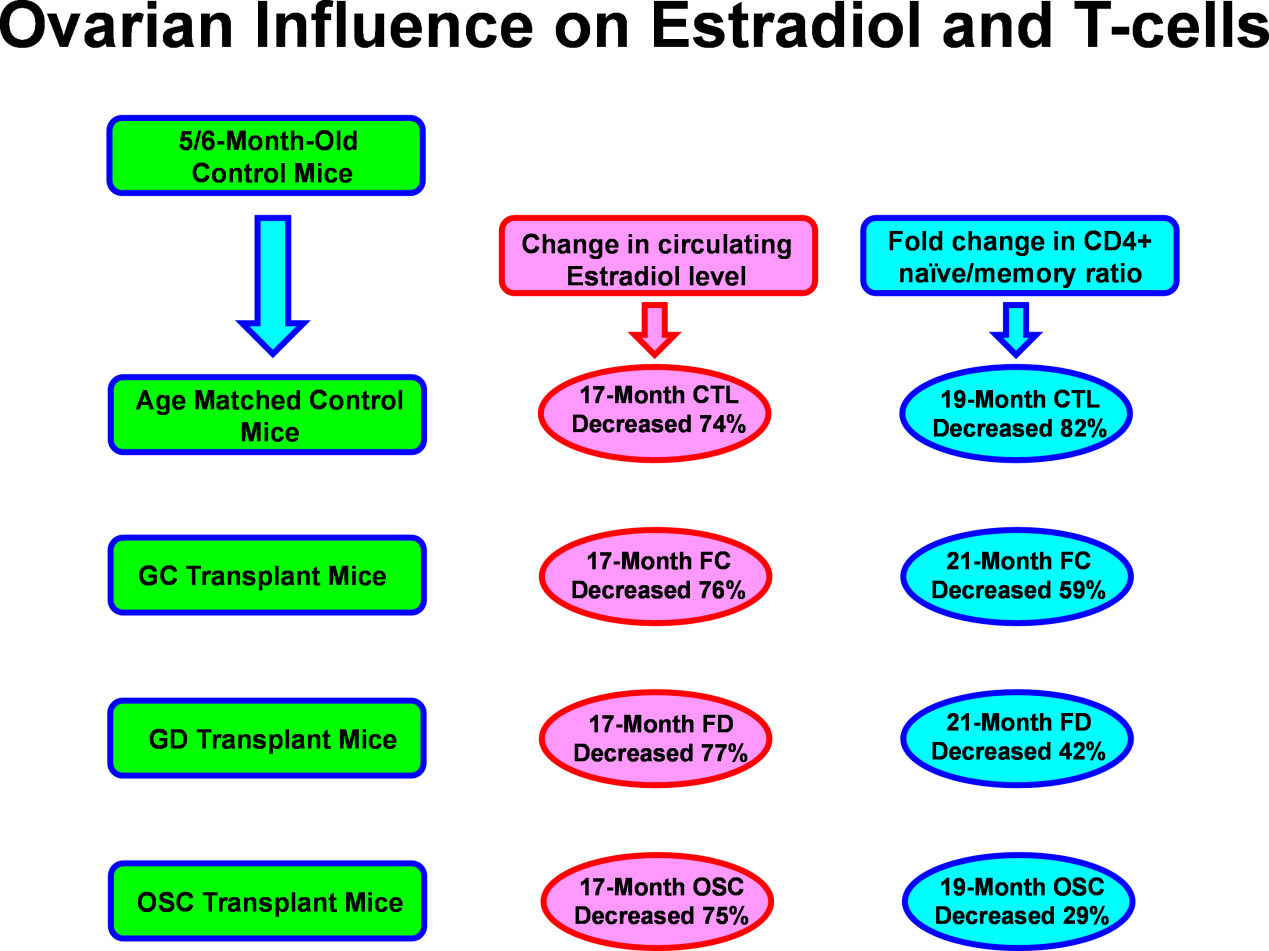
Aging and ovarian-associated changes in estradiol and T cell function. Estradiol remained low while T cell function improved with exposure to young ovarian tissue in aged female mice. FC, transplanted with follicle-containing young 60d ovaries at 13mo of age; FD, transplanted with follicle-depleted young 60d ovaries at 13mo of age; OSC, transplanted with isolated young 60d ovarian somatic cells at 13mo of age.

A prevailing view in mammalian aging is that estrogen represents the only important reproductive influence on health. The dramatic changes observed in the health of old mice that received new ovaries (33, **Figure 1**) could be easily dismissed as a simple restoration of circulating estrogen levels. Estradiol is the most widely cited hormonal influence on post-reproductive female health. The influence of estradiol is very different in post-reproductive females, compared with young females and the results of estrogen supplementation in post-reproductive females have historically been ambiguous. In a recent study replicated independently by three laboratories (36), 17-α-estradiol did not affect female life span when fed from 10 months of age. This is certainly not the same hormone as the more well-known 17-β-estradiol, but many of the health-associated effects of estrogen have been ascribed to 17-α-estradiol signaling (37). The beneficial effects of 17-α-estradiol are often thought to be due to actions independent of the classical estrogen receptor (38). Both 17a and 17b estradiol can alter adaptive immune T cell subsets, but 17-α-estradiol can suppress IFNα, in contrast to increased IFNα from 17-β-estradiol exposed mice, which may exacerbate the autoimmune inflammatory processes in females (39). T cells facilitate maternal immune tolerance of the fetus during pregnancy and may protect against autoimmune diseases (women are more prone to autoimmune diseases, 52). Estrogen therapy has been shown to have both positive and negative influences on many age-related pathologies in post-menopausal women and clearly does not possess the same influence in post-menopausal women as it does in young women.

While germ cell/follicle estrogen production is critical for reproduction, at menopause, germ cell-depleted ovaries still possess health-promoting attributes, as removal of the post-reproductive ovaries further increases rates of mortality. It has been documented that if the reproductively-senescent ovaries are absent in post-menopausal women, then the rate of all-cause mortality (including age-related diseases) increases (40, 41). If the processes influencing longevity are evolutionarily conserved, evidence from model organisms argues against an estrogen-only mechanism for health span extension in mammals. In both worms and flies, gonadal germ cells act in the adult to influence lifespan. In the hermaphroditic worm *C. elegans*, removal of the germ cells, while leaving the somatic gonad intact results in increased lifespan, but removal of the entire gonad, including the somatic tissue yields no change (51). Similar results are found in the fly, *D. melanogaster* (42). In both species, the somatic gonad promotes health span, whereas the germ components of the gonad act to suppress it.

Because of the notable differences in reproductive physiology between mice and *C. elegans* and *D. melanogaster*, any observed experimental differences are challenging to interpret. While the influence of the gonad on longevity does appear to be evolutionarily conserved in mammals (35), determining the role of germ cells, and importantly cyclic sex hormones in this phenomenon would be necessary to interpret more clearly our earlier results and would require separation of germ line and somatic gonadal tissues in a mammalian model and removing the germ cell influence from the ovaries.

Since the germ cells in the young, transplanted ovaries could be contributing more than just estradiol/cyclic hormones, we chemically depleted the germ cells/follicles from the young ovaries (using 4-vinylcyclohexene diepoxide) prior to transplantation. In this way we removed cyclic hormone production and any other signaling factors that may be produced by the germ cells/follicles. Chemically depleting the germ cells/follicles from young ovaries exposes the young mice to the toxic effects of VCD, which would make it difficult to separate the effects of loss of germ cells from the toxic effects of VCD. However, removing the ovaries from the toxin-treated mice and placing the VCD-treated ovaries in a non-VCD treated recipients avoids the complications associated with systemic VCD toxicity (43). Mice that received follicle-depleted young ovaries (FD) demonstrated the same or, in many cases better recovery of naïve T-cell function than that seen in mice that received young intact ovaries (with intact follicles-FC). To further separate this ovarian somatic function from the structure of the ovary, we isolated single somatic cells from young ovaries and injected them directly into the senescent ovaries of reproductively-senescent mice. Mice that received isolated young ovarian somatic cells (OSC) whilst retaining their old, senescent ovaries again demonstrated the same or, in most cases better recovery of naïve T-cell function than that seen in mice that received FC or FD young ovaries (**Table 1**). These observations further question the role of senescent ovaries in the decline of female health.

Removal of the follicles eliminated the possibility of the transplanted ovaries/cells from producing cyclic hormones (44). While the follicles direct cyclic hormone production, it is the somatic cells that produce the hormones, so while follicle depletion will remove cyclic hormone production, it is possible that acyclic hormone production could still occur in the young ovarian somatic cells (28). Previous work has documented that estradiol decreases with age/loss of ovarian function in mammals (45, 46). As expected in our mice, estradiol was decreased with age/loss of ovarian function. To explore this relationship further, we next looked at estradiol in the young ovary/cell transplant recipient groups (FC, FD and OSC). Estradiol levels in the FD and OSC groups were very low, as was seen in the age-matched control mice (28). Surprisingly, estradiol in the FC group also was low, not increased, although AMH was increased in this group (supporting the presence of primary or further advanced follicles) compared to the other transplant groups and the age-matched controls. Unlike the FD and OSC groups, the FC group had initially resumed irregular reproductive cycling post-transplantation, but were acyclic 4 months post-operatively. In humans, orthotopic heterotopic transplantation of ovarian tissue most often results in some restoration of endocrine function (47–50). The transplant recipient mice all had young ovarian tissues, but they also had an old hypothalamus and pituitary, which likely compromised hormone signaling and prevented a significant rise in estradiol, even with the ovarian potential to do so. This raises the possibility that the beneficial health influence of the young transplanted ovarian tissues may be independent of hypothalamic-pituitary-gonadal axis signaling.

## Summary

As has been seen previously, increased aging in female mice leads to decreased immune function and more specifically, to a decreased availability of naïve T-cell subsets and decreased estradiol signaling (21, 22). Increased exposure to young ovarian somatic tissues improved T-cell function in post-reproductive recipient mice and appeared to do so independent of changes in estradiol levels. Our results suggest that the production of naïve T-cells is improved in post-reproductive female mice by exposure to young ovarian tissue. An alternative explanation may be that the survival of naïve T-cells is extended. In our mice and in other studies, IL-7, which can prolong naïve T-cell longevity decreased with age, but increased significantly in transplant recipients (12, 14), suggesting that *preservation* rather than *restoration* was responsible for the increase in naïve T-cells. While the interactions between hormones and immune function are complex and support a correlation between endocrine function and immune response, the evidence presented here suggests the presence of additional, age and gender-specific influences on health and immune function. The molecular mechanisms behind the ovarian-dependent and estradiol-independent restoration of naïve T-cell function in post-reproductive female mice is currently being investigated further and holds promise for the future restoration of health in post-reproductive females and in females with early ovarian failure.

## Author contributions

JM conceived of the concept, designed the experiments, contributed to interpretation of the data, and contributed to writing the manuscript. TK, BB, NJ, KU and MW ran experiments, collected data, contributed to interpretation of the data, and contributed to writing the manuscript. SZ contributed to interpretation of the data, and contributed to writing the manuscript. All authors contributed to the article and approved the submitted version.
